# Biomarkers of success of anti-PD-(L)1 immunotherapy for non-small cell lung cancer derived from RNA- and whole-exome sequencing: results of a prospective observational study on a cohort of 85 patients

**DOI:** 10.3389/fimmu.2024.1493877

**Published:** 2024-12-12

**Authors:** Elena Poddubskaya, Maria Suntsova, Marina Lyadova, Daniil Luppov, Anastasia Guryanova, Vladimir Lyadov, Andrew Garazha, Maksim Sorokin, Anna Semenova, Vitaly Shatalov, Maria Biakhova, Alexander Simonov, Aleksey Moisseev, Anton Buzdin

**Affiliations:** ^1^ Institute of Personalized Oncology, I.M. Sechenov First Moscow State Medical University, Moscow, Russia; ^2^ Vitamed Clinic, Moscow, Russia; ^3^ Laboratory of Translational Genomic Bioinformatic, Moscow Institute of Physics and Technology, Dolgoprudny, Russia; ^4^ Oncology Center No. 1, Moscow City Hospital Named after S. S. Yudin, Moscow Healthcare Department, Moscow, Russia; ^5^ Department of Molecular Genetic Research, Endocrinology Research Center, Moscow, Russia; ^6^ Branch Campus of the Federal State Budgetary Educational Institution of Further Professional Education «Russian Medical Academy of Continuous Professional Education» of the Ministry of Healthcare of the Russian Federation, Novokuznetsk, Russia, Novokuznetsk, Russia; ^7^ Department of Research, Oncobox Ltd., Moscow, Russia; ^8^ Laboratory for Genomic Analysis of Cell Signaling Systems, Shemyakin-Ovchinnikov Institute of Bioorganic Chemistry, Moscow, Russia; ^9^ PathoBiology Group, European Organization for Research and Treatment of Cancer (EORTC), Brussels, Belgium

**Keywords:** immune checkpoint therapy, pembrolizumab, ipilimumab, nivolumab, non-small cell lung cancer, RNA sequencing, gene expression biomarker, personalized medicine

## Abstract

**Background:**

Immune checkpoint inhibitors (ICIs) treatment have shown high efficacy for about 15 cancer types. However, this therapy is only effective in 20-30% of cancer patients. Thus, the precise biomarkers of ICI response are an urgent need.

**Methods:**

We conducted a prospective observational study of the prognostic potential ofseveral existing and putative biomarkers of response to immunotherapy in acohort of 85 patients with lung cancer (LC) receiving PD-1 or PD-L1 targeted ICIs. Tumor biosamples were obtained prior to ICI treatment and profiled by whole exome and RNA sequencing. The entire 403 putative biomarkers were screened, including tumor mutation burden (TMB) and number of cancer neoantigens, 131 specific HLA alleles, homozygous state of 11 HLA alleles and their superfamilies; four gene mutation biomarkers, expression of 45 immune checkpoint genes and closely related genes, and three previously published diagnostic gene signatures; for the first time, activation levels of 188 molecular pathways containing immune checkpoint genes and activation levels of 19 pathways algorithmically generated using a human interactome model centered around immune checkpoint genes. Treatment outcomes and/or progression-free survival (PFS) times were available for 61 of 85 patients with LC, including 24 patients with adenocarcinoma and 27 patients with squamous cell LC, whose samples were further analyzed. For the rest 24 patients, both treatment outcomes and PFS data could not be collected. Of these, 54 patients were treated with PD1-specific and 7 patients with PD-L1-specific ICIs. We evaluated the potential of biomarkers based on PFS and RECIST treatment response data.

**Results:**

In our sample, 45 biomarkers were statistically significantly associated with PFS and 44 with response to treatment, of which eight were shared. Five of these (CD3G and NCAM1 gene expression levels, and levels of activation of Adrenergic signaling in cardiomyocytes, Growth hormone signaling, and Endothelin molecular pathways) were used in our signature that showed an AUC of 0.73 and HR of 0.27 (p=0.00034) on the experimental dataset. This signature was also reliable (AUC 0.76, 0.87) for the independent publicly available LC datasets GSE207422, GSE126044 annotated with ICI response data and demonstrated same survival trends on independent dataset GSE135222 annotated with PFS data. In both experimental and one independent datasets annotated with samples’ histotypes, the signature worked better for lung adenocarcinoma than for squamous cell LC.

**Conclusion:**

The high reliability of our signature to predict response and PFS after ICI treatment was demonstrated using experimental and 3 independent datasets. Additionally, annotated molecular profiles obtained in this study were made publicly accessible.

## Introduction

Immunotherapy by immune checkpoint inhibitors has changed the treatment landscape for many cancers in recent years. Unlike chemotherapy and targeted therapies, which target tumor cells directly, immunotherapy stimulates the patient's immune response or enhances the patient's ability to fight tumor cells. ICIs target regulators of immune checkpoints such as cytotoxic T-lymphocyte associated protein 4 (CTLA4), programmed cell death-1 (PD-1), or programmed death ligand 1 (PD-L1). Since the FDA approved a CTLA-4 inhibitor (ipilimumab) in 2011, six other ICIs have been approved by the FDA (1). Of these, three are PD-1 inhibitors (nivolumab, pembrolizumab, and cemiplimab) and three are PD-L1 inhibitors (atezolizumab, avelumab, and durvalumab). These ICIs are widely used in the daily practice of oncologists in the treatment of about 15 tumor types (2) and have shown high efficacy.

However, ICI treatment is only effective in 20-30% of cancer patients (3). Most patients do not respond to treatment or are resistant to treatment, which may be due to low infiltration by T cells, low tumor mutational burden (TMB), and poor antigenic presentation (3). Given the high cost of immunotherapy, effective identification and selection of potential responders has become a clinical challenge for the effective use of ICIs (4). There is an urgent need to develop and validate more accurate biomarkers to aid in the selection of patients for treatment with ICIs.

Several different types of predictive biomarkers have been developed to optimize the use of immunotherapy, including positive and negative predictive biomarkers to predict response or resistance to ICIs (5, 6), and side effect biomarkers to predict immune-related toxicity (7). Of these, three FDA-approved positive predictive biomarkers, PD-L1, microsatellite instability/defective mismatch repair (MSI/dMMR), and tumor mutational burden (TMB), are the most validated and clinically used (4). The use of these three FDA-approved biomarkers has played an important role in facilitating proper selection of patients for ICI treatment.

PD-L1 was the first prognostic biomarker for non-small cell lung cancer (NSCLC) approved by the FDA in 2015. PD-1 and PD-L1 belong to the family of immune checkpoint proteins. Their interaction plays a key role in regulating the immune system, ensuring that it is activated only at the right time to minimize excessive inflammation and autoimmune responses. PD-L1 is expressed on a variety of normal and immune cells such as dendritic cells, activated T and B lymphocytes and macrophages. However, cancers have also adopted this PD-1/PD-L1 interaction mechanism by expressing PD-L1 on the surface of tumor cells. Binding of tumor PD-L1 to PD-1 on T cells results in attenuation or inhibition of T cell activity, which helps tumor cells escape immune surveillance (8).

In turn, blocking the interaction between PD-L1 and PD-1 allows T cells to be reactivated and their activity against tumor cells to be enhanced. Since the number of tumor cells expressing PD-L1 greatly influences its ability to suppress immunogenicity and further determines the efficacy of PD-L1 and PD-1 blockade with ICI, PD-L1 expression on tumor cells is a predictive biomarker for ICI therapy (4). Despite its most widespread use, PD-L1 has low diagnostic accuracy in general, with particularly low negative predictive value. For example, it has been reported that up to 20% of patients with PD-L1-negative tumors benefit from PD-1/PD-L1 antibodies (9). In addition, PD-L1 expression is regulated in time and space (10) and may be altered by prior therapeutic treatment (11). The combination of these factors limits the predictability of PD-L1 in certain circumstances.

FDA approval and validation of MSI/dMMR was the second FDA-approved prognostic biomarker for pembrolizumab treatment of adult and pediatric patients with unresectable or metastatic solid tumors in 2017. The approval of pembrolizumab for the treatment of MSI-H (MSI-high)/dMMR cancer was based on evidence of efficacy from five clinical trials (12). This approval is the first drug approved for the treatment of solid tumors in general based on a generic biomarker rather than a specific tumor type. Tumors with a defective DNA mismatch repair (dMMR) system accumulate thousands of mutations throughout the genome. Because short tandem repeats are particularly susceptible to mismatch errors, dMMR-induced hypermutations are most often localized in microsatellite regions (short stretches of DNA 1-6 nucleotides long), a condition defined as microsatellite instability (MSI). MSI is a result and marker of dMMR (4). Tumors with dMMR will also have more mutations in non-MSI regions throughout the genome and are expected to have more neoantigens compared to tumors with intact MMR (13). However, this genetic abnormality is relatively rare in lung cancers being characteristic for colorectal, cervical, and ovarian tumors (14).

TMB is a measure of the number of gene mutations that can be reported as the total number of nonsynonymous somatic mutations in the tumor exome (15) or per megabase (16). TMB was approved for pembrolizumab for the treatment of adult and pediatric patients with unresectable or metastatic solid tumors. Foundation One CDx assay was also approved as a companion diagnostic test (4). A high number of mutations in somatic exonic regions will lead to an increase in neoantigen production, some of which are immunogenic, and could then be recognized by T cells, resulting in improved antitumor immune responses. Consequently, patients with high TMB likely produce more intensified immune responses and are more sensitive to ICI treatments (4).

In addition, a number of emerging biomarkers including various gene signatures have been proposed with the goal of finding more efficient and accurate biomarkers suitable for a broader population of tumor patients, including immunologically cold tumors (4). Additionally a number of signaling molecules, such as cytokines expressions were reported as a putative biomarkers of ICI response (17, 18).

Here we conducted a prospective observational study of the prognostic potential of several existing and putative biomarkers of response to immunotherapy in a cohort of 85 patients with lung cancer (LC) receiving PD-1 or PD-L1 targeted immune checkpoint inhibitors (ICIs). Tumor biosamples were obtained prior to ICI treatment and profiled by whole exome and RNA sequencing. The entire 403 putative biomarkers were screened, including tumor mutation burden (TMB) and number of cancer neoantigens, 131 specific HLA alleles, homozygous state of 11 HLA alleles and their superfamilies; four gene mutation biomarkers, expression of 45 immune checkpoint genes and closely related genes; for the first time, activation levels of 188 molecular pathways containing immune checkpoint genes and activation levels of 19 pathways algorithmically generated using a human interactome model centered around immune checkpoint; additionally, three existing signatures for ICI response prediction were assessed on our dataset. Treatment outcomes and/or progression-free survival (PFS) times were reported in 61 patients with lung cancer, including 24 patients with adenocarcinoma and 27 patients with squamous cell lung cancer. Of these, 54 patients were treated with PD1-specific and 7 patients with PD-L1-specific ICIs. We evaluated the potential of biomarkers based on PFS and RECIST treatment response data. In our sample, 45 biomarkers were statistically significantly associated with PFS and 44 with response to treatment, of which eight were shared. Five of these (*CD3G* and *NCAM1* gene expression levels, and levels of activation of Adrenergic signaling in cardiomyocytes, Growth hormone signaling, and Endothelin molecular pathways) were used to construct a signature that showed an AUC of 0.73 and HR of 0.27 (p=0.00034) on the experimental dataset. This signature was also reliable (AUC 0.76) for the independent publicly available LC dataset GSE207422 annotated with ICI response data. In both datasets, the signature worked better for lung adenocarcinoma than for squamous cell LC. All molecular profiles obtained in this study and their clinical annotations are in the public domain and can be freely used by the scientific community. 

## Materials and methods

### Patient biosamples

The study was designed and conducted in accordance with the ethical principles of the Declaration of Helsinki. The local ethical committee at the Vitamed Clinic, Moscow, approved the study design and public presentation of its results as a research paper; date of approval: 12 March 2019. All biosamples were formalin-fixed, paraffin-embedded (FFPE) solid tumor blocks obtained from primary or metastatic tumor sites and evaluated by a pathologist, with at least 60% cancer cells.

The patients included in this study received PD(L)1 specific ICI therapy within the frameworks of clinical trials NCT03777657 (19), Oncobox (NCT03724097), CheckMate 078 (20), and (21) CheckMate 817. All patients whose biosamples were included in the present study had previously signed written informed consents to participate in the observational clinical investigation and to have their biosamples profiled by sequencing using the Illumina HiSeq3000 or Illumina NextSeq550 next-generation sequencing platforms. The patients also agreed to the publication of depersonalized WES/RNAseq profiles of their cancer samples, as well as the publication of study results in the form of gene activity profiles associated with age, gender, and diagnosis.

All biosamples were collected prior to the treatment with ICI PD(L)1 therapeutics, which was the next line of treatment. Where possible, progression free survival times and tumor response statuses according to RECIST criteria were collected (**Supplementary Table 1**). The patients whose tumor response status was defined as *Complete Response* (CR) or *Partial Response* (PR) were categorized as the treatment responders. The patients whose RECIST treatment outcomes were *Stable Disease* (SD) or *Progressive Disease* (PD) were considered as the non-responders to treatment.

### RNA sequencing

RNA sequencing was performed at the Laboratory of Clinical Genomic Bioinformatics, Sechenov First Moscow State Medical University, as previously described (22, 23). Library construction and ribosomal RNA depletion were carried out using the KAPA RNA Hyper with rRNA Erase (HMR only) kit. Library concentrations were measured with the Qubit dsDNA HS Assay kit (Life Technologies) and quality was assessed using the Agilent Tapestation (Agilent). RNA sequencing was performed on an Illumina NextSeq 550 system for single-end sequencing with a 50 bp read length, generating at least 30 million raw reads per sample. Data quality was checked using Illumina SAV, and de-multiplexing was performed with Illumina Bcl2fastq2 v2.17 software.

### Processing of RNA sequencing data

RNA sequencing FASTQ files were processed using the STAR aligner (24) in "GeneCounts" mode with Ensembl human transcriptome annotation (Build version GRCh38, transcript annotation GRCh38.89). Ensembl gene IDs were converted to HGNC gene symbols using the Complete HGNC dataset (https://www.genenames.org/, database version from July 13, 2017). In total, expression levels were determined for 36,596 genes with HGNC identifiers. RNA-seq data normalization was performed using the DESeq2 (25), and a pseudo-count of 1 was added to the normalized counts.

### Whole exome DNA sequencing

Whole exome DNA sequencing (WES) was performed as previously described (26). DNA was extracted from FFPE tissue samples using the AnaPrep FFPE DNA extraction kit, and whole exome DNA was captured from total genomic DNA using the SeqCap EZ System from NimbleGen, following the manufacturer’s instructions. Briefly, genomic DNA was sheared, size-selected to approximately 200–250 base pairs, and the ends were repaired and ligated to specific adapters and multiplexing indexes. The fragments were then incubated with SeqCap biotinylated DNA baits followed by LM-PCR, and the RNA-DNA hybrids were purified using streptavidin-coated magnetic beads. The RNA baits were then digested to release the targeted DNA fragments, followed by a brief amplification of 15 or fewer PCR cycles. Sequencing was performed on an Illumina NextSeq 550 for a paired-end 150 run. Data quality was checked using Illumina SAV, and demultiplexing was performed with the Illumina Bcl2fastq2 v2.17 program.

### Processing of WES data

For WES data analysis, the GATK somatic mutation calling pipeline was utilized. Reads were aligned to the human genome version 38 using BWA mem v0.7.17 software (27) with the following non-default parameters: −k 15, −r 2. The remaining pre-processing steps were identical to those described for the RNAseq pipeline, except that reads splitting and mapping quality editing steps were omitted.

For mutation calling, GATK4 Mutect2 (28) software was used simultaneously for tumor and matched normal samples, supplied with the same dbSNP and indel databases, regions, and PCR model. Subsequent post-processing steps included filtering with GATK4 FilterMutectCalls and annotation with ANNOVAR. All tri- or more allelic sites were excluded from further analyses, as such mutations were not annotated using ANNOVAR and were not included in TMB calculation. For managing parallel computational tasks, GNU parallel software was employed.

### Construction of gene-centric molecular pathways

The gene-centric molecular pathways specifically centered around gene product of interest were algorithmically reconstructed as previously reported in (29). Briefly, a model of the human interactome was constructed using the OncoboxPD collection of human molecular pathways (30) as a knowledge base of molecular interactions. Gene composition and nodal pathway interactions were extracted and cataloged. All pathway graphs were merged based on overlapping gene products. Only the gene products were included that formed a connected network, *i.e.* there was a link between each pair of gene products.

For each of the specific gene products under analysis, algorithmic molecular pathways were constructed using gene products of interest as the central nodes. The following types of interactions were considered: "activation", "coupling", "inhibition", "phosphorylation", "dissociation", "repression", "dephosphorylation", "binding/association", and "ubiquitination".

### Pathway activation analysis

Functionally annotated structures of molecular pathways were extracted from online OncoboxPD database (30) and the enclosed bioinformatic instruments were used to assess the pathway activation levels (PALs) using experimental data.

The PAL values were calculated according to the following formula (31):


PALp=∑ARRnp·BTIFn·ln(CNRn),

where *CNRn* (case-to-normal ratio) is the ratio of gene *n* expression level in the sample under investigation to the mean geometrical expression level in the group of control samples. The Boolean flag *BTIFn* (beyond tolerance interval flag) is zero when the *CNRn* value has not passed the significance criterion: when the difference with the control group of samples is not significant, where *p*>0.05. *ARRn,p* (activator/repressor role of gene *n* in pathway *p*) is the discrete value that equals to −1 when gene product *n* is a repressor of pathway p; 1, when gene product *n* is an activator of pathway p; 0, when gene product *n* has both activities of an activator and of a repressor of pathway p; 0.5 and −0.5, respectively, when gene product *n* is rather an activator or repressor of pathway *p*.

Each profile was normalized on a control group of RNA sequencing profiles previously obtained for healthy human lung tissues by the same research group using the same sequencing equipment and protocols (22).

For the calculation of PALs we used DESeq2 normalized counts which were processed using OncoboxPD web tool (30) using built-in panel of normal lung tissues as the reference.

### HLA calling

HLA-A, HLA-B, HLA-C, HLA-DPB1, HLA-DQB1, and HLA-DRB1 alleles were assessed for all WES samples. HLA allele calling for both classes of MHC was done via xHLA software version 1.2 (32). HLA superfamilies were assigned according to Sidney et al. (33).

### Statistical biomarker analysis

ROC AUC value was calculated for each putative biomarker to assess its ability to distinguish between responders and non-responders to treatment with PD(L)1 specific ICI therapeutics. AUC calculations were performed using ‘pROC’ R package (v1.18.5) (34).

Additionally, Mann–Whitney U test was performed between the groups of responders and non-responders for numerical markers and Fishers’ exact test for categorical markers.

Survival analysis was performed using Cox regression model. For categorical markers (such as the presence of HLA alleles or specific mutations) model was fit by categorical explanatory variable. For numerical markers (such as gene expressions and PALs) numerical explanatory variables were translated to categorical using specific thresholds calculated by optimizing HR provided that in each group must have at least 30% of the sample size. After threshold determination, a categorical value was assigned to each patient and then used to fit Cox regression model. To fit Cox models and plot Kaplan Meier curves, ‘survminer’ (v0.4.9) and ‘survival’ (v3.5-5) (35) R packages were used.

### Assessment of diagnostic signatures

Tumor Immune Dysfunction and Exclusion (TIDE) signature (36) was quantitatively assessed using TIDEpy library (v. 1.3) (37) in the NSCLC mode using TPM values of genes.

T cell-inflamed gene expression profile (GEP) (38) signature was assessed using weighted sum of normalized by housekeeping genes log2 TPM expression values according to (39).

Integral GEP + TMB signature was assessed as a categorical marker between the groups of patients with TMB^high^ +GEP^high^ and TMB^low^+GEP^low^. “High” TMB status was determined as TMB exceeding 10 non-synonymous mutations per megabase of coding genomic sequence and “high” GEP status was determined as GEP value exceeding optimized threshold calculated for PFS analysis.

### Principal component analysis

Principal component analysis (PCA) was performed using TPM gene expression values with scikit-learn (v. 1.5.1) software.

## Results

This study was designed to establish whole-exome (WES) and RNA sequencing profiles of non-small cell lung cancer (NSCLC) biosamples obtained from patients prior to immunotherapy with PD-1 or PD-L1 specific ICI therapeutics. In this longitudinal study, progression-free survival (PFS) times and RECIST criteria-based treatment outcomes for the patients under analysis were recorded and analyzed. We explored totally 403 putative molecular biomarkers including tumor mutation burden (TMB) and number of cancer neoantigens, characteristic gene signatures, 131 specific HLA alleles, homozygous state of 11 HLA alleles and their superfamilies; four gene mutation biomarkers, expression of 45 immune checkpoint genes and closely related genes and three previously published signatures. For the first time, we assessed biomarker potential of the activation levels of 188 molecular pathways containing immune checkpoint genes and activation levels of 19 pathways algorithmically generated using a human interactome model centered around immune checkpoint genes. Based on the best biomarkers detected, a synthetic biomarker signature was developed with the optimal PFS and RECIST survival predictive value that was further validated on an independent clinically annotated lung cancer immunotherapy molecular dataset GSE207422.

### Clinical cohort and biosamples

The cohort under analysis included stage IV NSCLC patients with available formalin-fixed, paraffin-embedded (FFPE) tumor tissue biosamples obtained before the treatment with PD-1-specific or PD-L1-specific ICI immunotherapeutic drugs, or both. Treatment with ICI therapeutics was the next line after obtaining tissue biomaterials. The biomaterials were biopsies or surgical materials obtained for either primary or metastatic tumors (**Supplementary Table 1**). Initially, 85 patients were included into the investigation. However, for 24/85 patients no further ICI treatment outcomes could be obtained. Thus, 61 patients where either RECIST response status or PFS time, or both were registered, were included in further analysis (**Supplementary Table 1**).

The patients were 20 women (47-79 years old, mean 59 y.o.) and 41 men (41-75 years old, mean 62 y.o.), **Table 1**, **Supplementary Table 1.**

**Table 1:** Summary statistics of the Oncobox NSCLC Immunotherapy cohort.

	Total NSCLC	Lung Adenocarcinoma	Lung Squamous Cell Carcinoma	Other histotypes
# of patients	61	24	27	10
# of female patients	20	13	2	5
# of male patients	41	11	25	17
Patients age range	41-79	41-77	45-79	50-74
# of responders	15	3	8	4
# of metastatic samples	12	8	1	3
# of patients with anti-PD-1 therapy	54	23	26	5
# of patients with anti-PD-L1 therapy	7	1	1	5
# of patients with anti-CTLA4 therapy	11	4	6	1
Median PFS time, months	6	8.5	6	4
# of events	41	16	20	5

**Figure 1:**
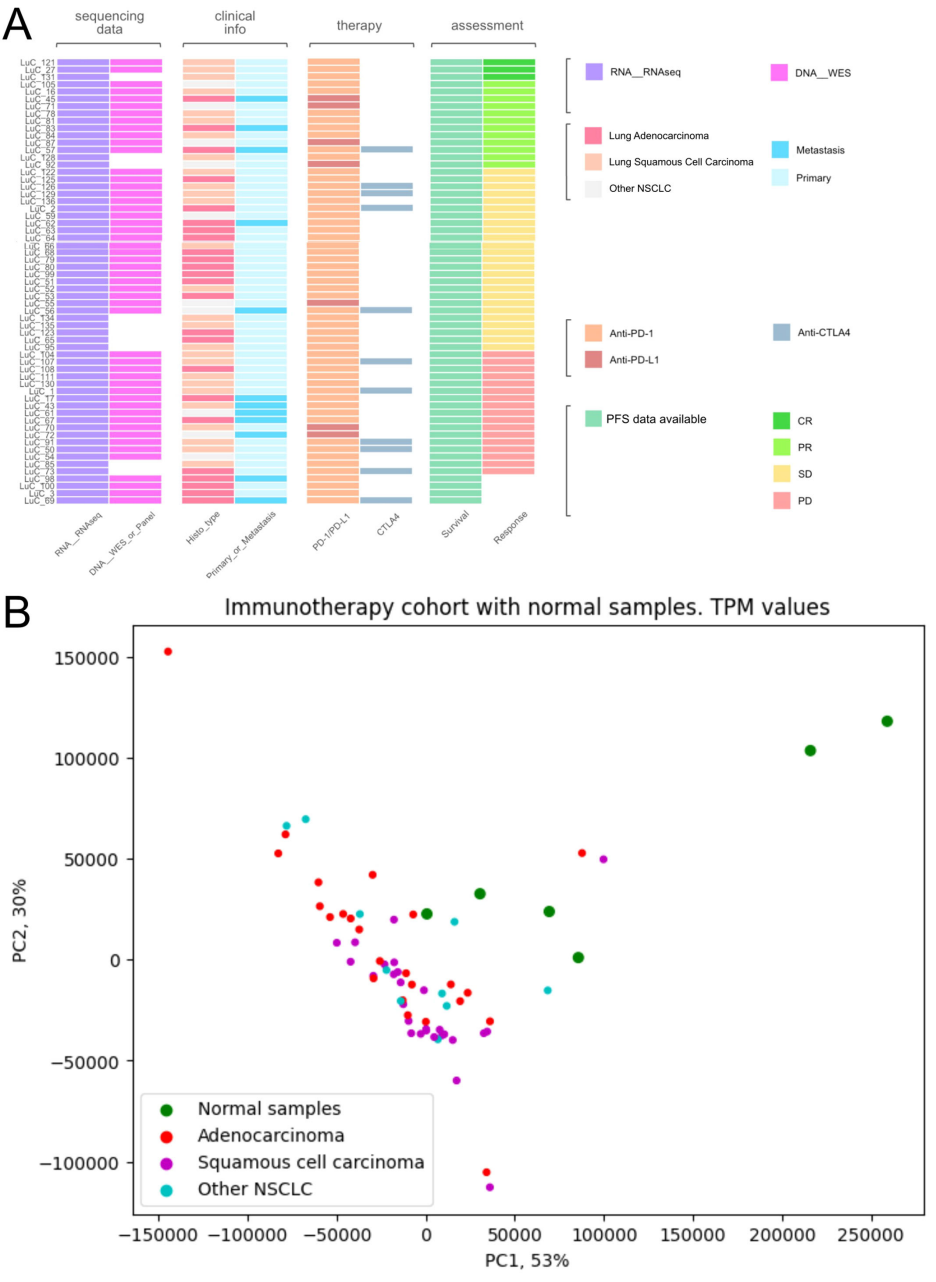
Characteristics of biosamples of the experimental NSCLC cohort. **(A)** Technical characteristics of biosamples under analysis. Sample IDs are given on the left. Color markers indicate availability of the RNAseq, WES profiles; tumor Histotypes established by pathologists; primary or metastatic origin of tumor samples; treatment with PD-1 or PD-L1 ICI therapeutics; treatment with CTLA4 ICI therapeutics; Availability of PFS data and availability of RECIST treatment response data. More detailed characteristics are given in **Supplementary Table 1**. **(B)** Principal component analysis (PCA) plot of RNA sequencing profiles in the experimental NSCLC cohort in comparison with the healthy lung tissue controls. Dot color indicates tissue type.

Among them, 27 patients were diagnosed with squamous cell carcinoma, 24 with adenocarcinoma, 9 had mixed phenotype, and one with neuroendocrine cancer (**Figure 1**, **Supplementary Table 1**). In 12 cases, the biosamples were isolated from metastatic sites, whereas the remaining 49 biosamples represented the primary tumors.

RNA sequencing (RNAseq) data were obtained for all 61 samples under analysis, whereas whole exome sequencing (WES) data could be obtained for 51/61 samples because DNA of sufficient quality for sequencing could not be isolated from 10/61 biosamples (**Supplementary Table 1**).

The RNA sequencing results are freely accessible through GEO repository with ID GSE274975.

Twenty-six patients received PD-1 or PD-L1 (PD(L)1)-specific ICI therapeutics as the monotherapy, whereas the others received combination therapies (**Supplementary Table 1**). Fifty-four patients received PD-1-specific and seven patients – PD-L1-specific ICI therapeutics (**Supplementary Table 1**). Eleven patients received combined treatment with (PD(L)1)-specific and CTLA4-specific ICI therapeutics (**Figure 1**, **Supplementary Table 1**).

The data on RECIST response status and PFS times are outlined in **Supplementary Table 1.**

The summary statistics of the entire patient cohort is given in **Table 1**.

### Assessment of putative molecular biomarkers

**Figure 2:**
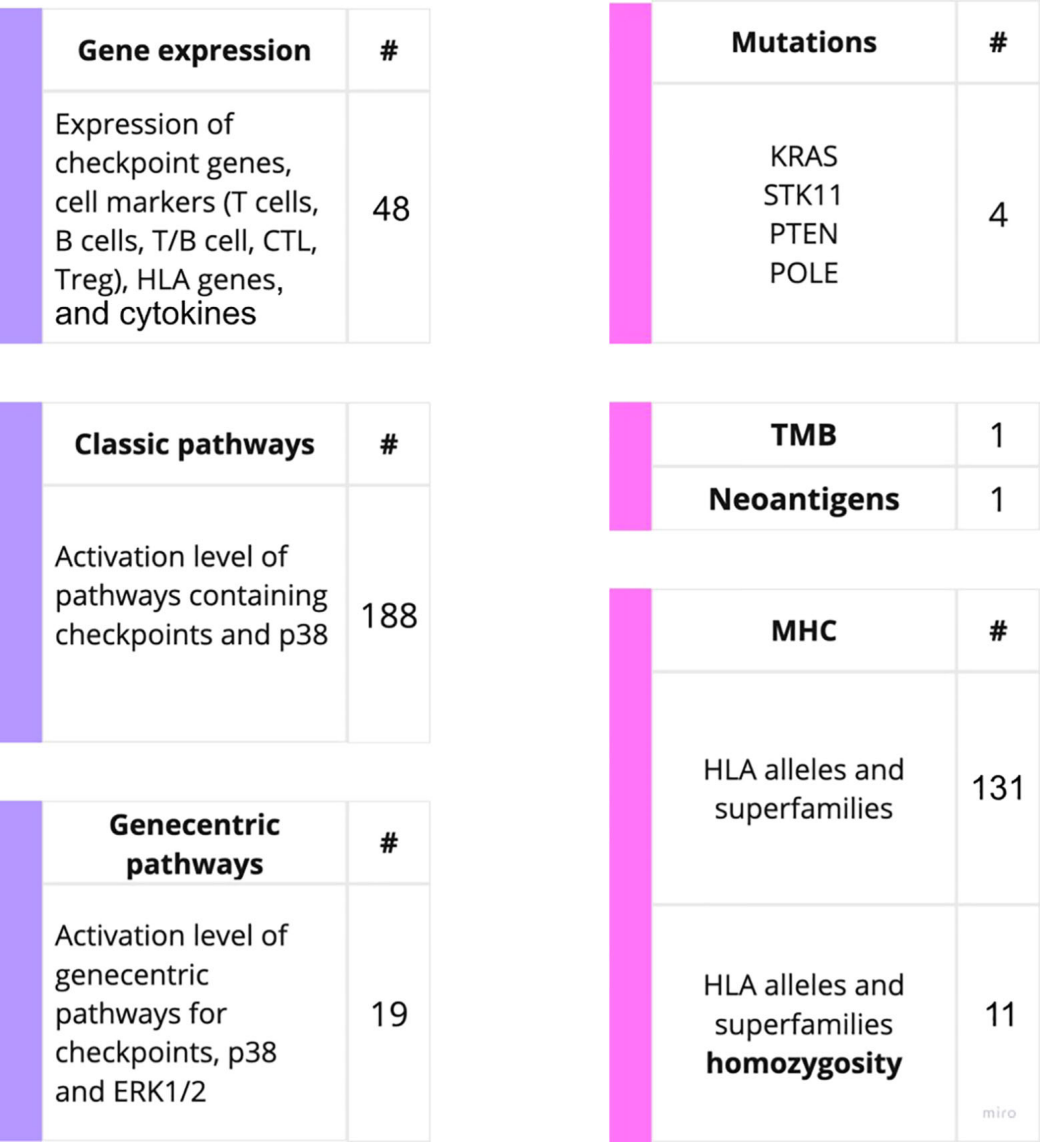
Outline of the putative PD(L)1 ICI NSCLC response biomarkers investigated in this study. The putative biomarkers used were assessed using RNA sequencing (left) or WES data (right).

On our experimental sampling, we then assessed the predictive capacity of totally 403 putative molecular ICI therapy biomarkers in relation to either RECIST response or PFS times for the treatment with PD(L)1 ICI therapeutics (**Figure 2**). For these analyses, we considered patients who demonstrated RECIST “complete response” and “partial response” as the *treatment responders*, whereas RECIST “progressive disease” and “stable disease” outcomes were attributed to the *non-responders to treatment*.

The biomarkers under analysis included tumor mutation burden (TMB) and number of cancer neoantigens, presence of 131 MHC (*HLA*) alleles identified by WES, homozygous state of *HLA* genes and their superfamilies which were previously associated with response to PD(L)1 ICI therapeutics in melanoma (40). This list also included presence of mutations in *KRAS, STK11, POLE* and *PTEN* genes previously associated with the sensitivity to ICI treatments (4). These results are summarized on **Supplementary Table 2.**

Additionally, immunohistochemical status of PD-L1 was assessed for 22 patients (12 positives, 10 negatives) for which these records were available. This biomarker demonstrated expected trend, yet the results of ICI response prediction (AUC = 0.72, Fisher’ exact test p = 0.14) and survival analysis (HR = 0.66, p = 0.42) appeared insignificant.

For the gene expression biomarkers, we assessed 45 transcriptional levels of known immune checkpoint genes or relevant genes playing major roles in p38 signaling along with expression levels of cytokines previously reported as ICI response biomarkers (**Supplementary Table 3**). In addition, for the first time, we assessed the activation levels of intracellular molecular pathways as the putative ICI response biomarkers. The pathway activation level (PAL) is the metric that reflects the extent of up/downregulation of a pathway in tumor samples under analysis compared to the corresponding normal samples. Positive PAL indicates upregulation of a pathway, zero PAL means no changes in pathway activation, and negative PAL means downregulation (30). PAL values were previously reported as the biomarkers for various types on non-ICI targeted therapeutics in human cancers such as ramucirumab, trastuzumab, bevacizumab, cetuximab and sorafenib (41–46). In a recent case report, extreme activation of a PAL of PD-1 signaling cascade along with the high tumor infiltration by T-cells was a main reason to successfully prescribe off-label PD-1 specific immunotherapy to a IV stage chemoresistant gastric cancer patient (26).

Thus, we assessed activation levels of 188 human molecular pathways containing immune checkpoint genes or p38 MAPK proteins which were found relevant to ICI responsiveness in previous research (47). Furthermore, based on the previous human cancer cell interactome models (48, 49) we algorithmically reconstructed 19 additional molecular pathways specific for the immune checkpoint genes, and used their activation levels as the putative biomarkers (**Supplementary Table 3**). The following immune checkpoint genes were considered: *LILRB4, TIGIT, LAG3, HAVCR2, PDCD1, CTLA4, CD28, TNFRSF9, ICOS, CD56, CD226, CD274, CD80, CD86, TNFSF9, ICOSLG, VSIR, LILRB2*, and *CD276.* Additionally, the expression of the number of cytokines were assessed in concordance with previous research. The cytokines-related genes being considered includes: *CXCL8, CXCL10, CXCL11, IL2, IL6, TNF* (18, 50).

Finally, we also assessed the biomarker potential of the previously published gene signatures *T-cell inflamed gene expression profile* (GEP), *T-cell dysfunction and exclusion gene signature* (TIDE), and TMB+GEP signatures that have been reported as the emerging ICI response biomarkers (4, 51).

Among them, the highest predictive capacity was demonstrated by GEP signature with hazard ratio (HR) = 0.31 (p= 0.0011) for PFS analysis, however, it could not differentiate the groups of treatment responder and non-responder patients (AUC = 0.56). Combination of TMB with GEP signature demonstrated non-statistically significant association with PFS and poor AUC value (AUC = 0.55). In turn, TIDE signature also showed non-statistically significant association with PFS and poor AUC value (AUC = 0.55).

**Figure 3:**
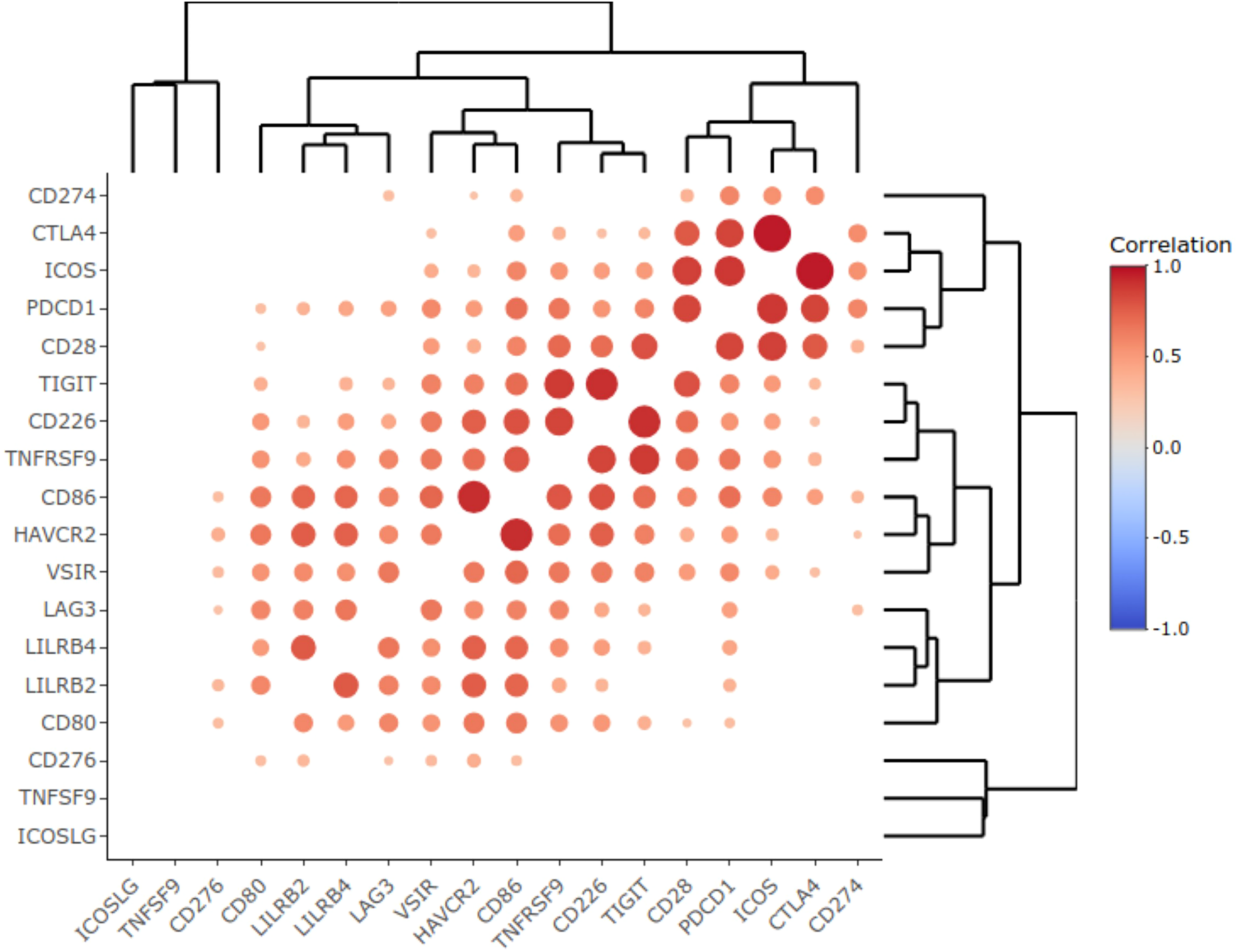
Correlation heat map representing statistically significant Pearson correlation coefficients obtained for the expression of immune checkpoint genes in the experimental NSCLC cohort. Color reflects correlation coefficients (see color scale) and dot size corresponds to the -log10(*p-value*) of the Pearson correlation test.

Interestingly, in our NSCLC sampling we observed correlated transcriptional activities of some immune checkpoint genes that formed clear-cut clusters on the correlation dendrogram (**Figure 3**). In particular, we found clustered expression of *CD80, LILRB2, LILRB4* and *LAG3* genes (cluster 1), *VSIR, HAVCR2, CD86, TNFRSF9, CD226* and *TIGIT* genes (cluster 2), and, most importantly, coordinated expression of *CD28*, *PDCD1, ICOS*, and *CTLA4* genes (cluster 3), **Figure 3**.

On the whole NSCLC sampling we found 45 putative biomarkers that were statistically significantly associated with survival times (PFS thresholds were hazard ratio (HR) greater than 2.5 or less than 0.4, and *p-value<*0.05),**Supplementary Table 3**. Among them, there were 11 WES-based putative biomarkers (listed from greater to lower significance): the presence of HLA alleles *DP-B1-104-01, DQ-B1-05-04, B-49-01, A-02-05, DQ-B1-06-11, DP-B1-02-01, DQ-B1-03-01, DQ-B1-06-03, DR-B1-13-01, A-26-01*, and *B-44-02*.

Seven PFS biomarkers were related to transcriptional activities of individual genes (*NCAM1, CD3G, ICOS, CTLA4, CD3D, CD3E, CXCL11*,and *FOXP3*).

Other seven PFS biomarkers were related to the algorithmically reconstructed gene-centric pathways (built around proteins CD86, TIGIT, CD226, CD80, CD274, PDCD1, and PDCD1LG2)

Finally, the remaining 19 PFS biomarkers were the activation levels of previously catalogued molecular pathways (from greater to lower significance) *(1): Co-stimulatory signal during T-cell activation main pathway, (2) Endothelins main pathway, (3) Co-stimulation by the CD28 family main pathway, (4) The 41bb-dependent immune response main pathway, (5) Adrenergic signaling in cardiomyocytes main pathway, (6) STAT3 pathway (growth arrest and differentiation), (7) Tumor infiltration pathway, (8) Rheumatoid arthritis main pathway, (9) Growth hormone signaling pathway (gene expression via SRF, ELK1, STAT5B, CEBPD, STAT1, STAT3), (10) PD-1 signaling main pathway, (11) Growth hormone signaling pathway, (12) TRAF pathway, (13) FLT3 signaling pathway, (14) RANK signaling in osteoclasts pathway, (15) Dopaminergic synapse main pathway, (16) FLT3 signaling pathway (transcription via ELK3, MAPK12, CREB3, STAT2), (17) ErbB family pathway, (18) GPCR pathway (gene expression via JUN, NFKB2, ELK1, SRF, FOS, CREB3)*, and *(19) IL10 pathway*. Of note, all of these pathways were the positive treatment response biomarkers as could be judged by their HR values (**Supplementary Table 3**).

On the other hand, when the biomarkers were selected based on their capacity to distinguish between RECIST responders and non-responders, 44 putative biomarkers were found (**Supplementary Table 3**). To identify them we applied two criteria: Mann-Whitney test *p-value<*0.05 for distinguishing the treatment responders and non-responders and ROC AUC > 0.62. AUC, area under ROC-curve, is a metric summarizing specificity and sensitivity of a biomarker in two-class separation. It varies between 0.5 and 1, and good-quality clinical biomarkers typically demonstrate AUC higher than 0.7 (17).

The threshold of AUC>0.62 was selected to identify 10% of top biomarkers by RECIST response.

Among them, only three WES-based biomarkers were found (sorted from greater to lower significance): HLA alleles *A24* and *DP-B1*, and mutation in *POLE* gene.

The following seven gene expression biomarkers were found (from top to bottom): *PDCD1, CTLA4, CD274, IL2, IL2RA, NCAM1, CD3G*, and *CD86*.

For gene-centric pathways, two putative biomarkers were found: for pathways built around MAPK14 and MAPK11 proteins.

Finally, the biggest number of such RECIST response biomarkers represented the previously established molecular pathways (29 biomarkers): *(1) Growth hormone signaling pathway (gene expression via SRF, ELK1, STAT5B, CEBPD, STAT1, STAT3), (2) Prolactin signaling main pathway, (3) Endothelins main pathway, (4) Adrenergic signaling in cardiomyocytes main pathway, (5) Growth hormone signaling pathway, (6) S1P2 main pathway, (7) CDO in myogenesis main pathway, (8) Pertussis main pathway, (9) TGF-beta pathway (transcription-arrested growth, apoptosis), (10) TGF-beta pathway (transcription-cell growth and mobility and angiogenesis), (11) Chemokine pathway (cell activation), (12) Activation of the AP1 family of transcription factors main pathway, (13) CD40-CD40L signaling main pathway, (14) Oocyte meiosis main pathway, (15) cAMP pathway (cytokine production), (16) Activation of PPARGC1A PGC1-alpha by phosphorylation main pathway, (17) DSCAM interactions main pathway, (18) cAMP pathway, (19) RANK signaling in osteoclasts pathway (expression of osteoclastic genes via JUN, NFAT5, NFKB2, MITF, FOS), (20) RAC1 signaling main pathway, (21) Chemokine pathway (gene expression and apoptosis via ELK1), (22) MAP kinase signaling main pathway, (23) Stathmin and breast cancer resistance to anti-microtubule agents main pathway, (24) IL10 pathway, (25) ErbB family pathway (gene expression via JUN, FOS, ELK1), (25) RIG1-like receptor signaling main pathway, (26) IL10 pathway (IL 10-responsive genes: transcription of BCLXL, Cyclin D1, D2, D3, Pim1, c-Myc, P19, INK4D via STAT3), (27) IL10 pathway (inflammatory cytokine gene expression via STAT3), (28) MAPK signaling main pathway, (29) Rap1 signaling main pathway*. Interestingly, as for the PFS biomarkers, all of these pathways except for Oocyte meiosis main pathway were the positive treatment response biomarkers.

**Figure 4:**
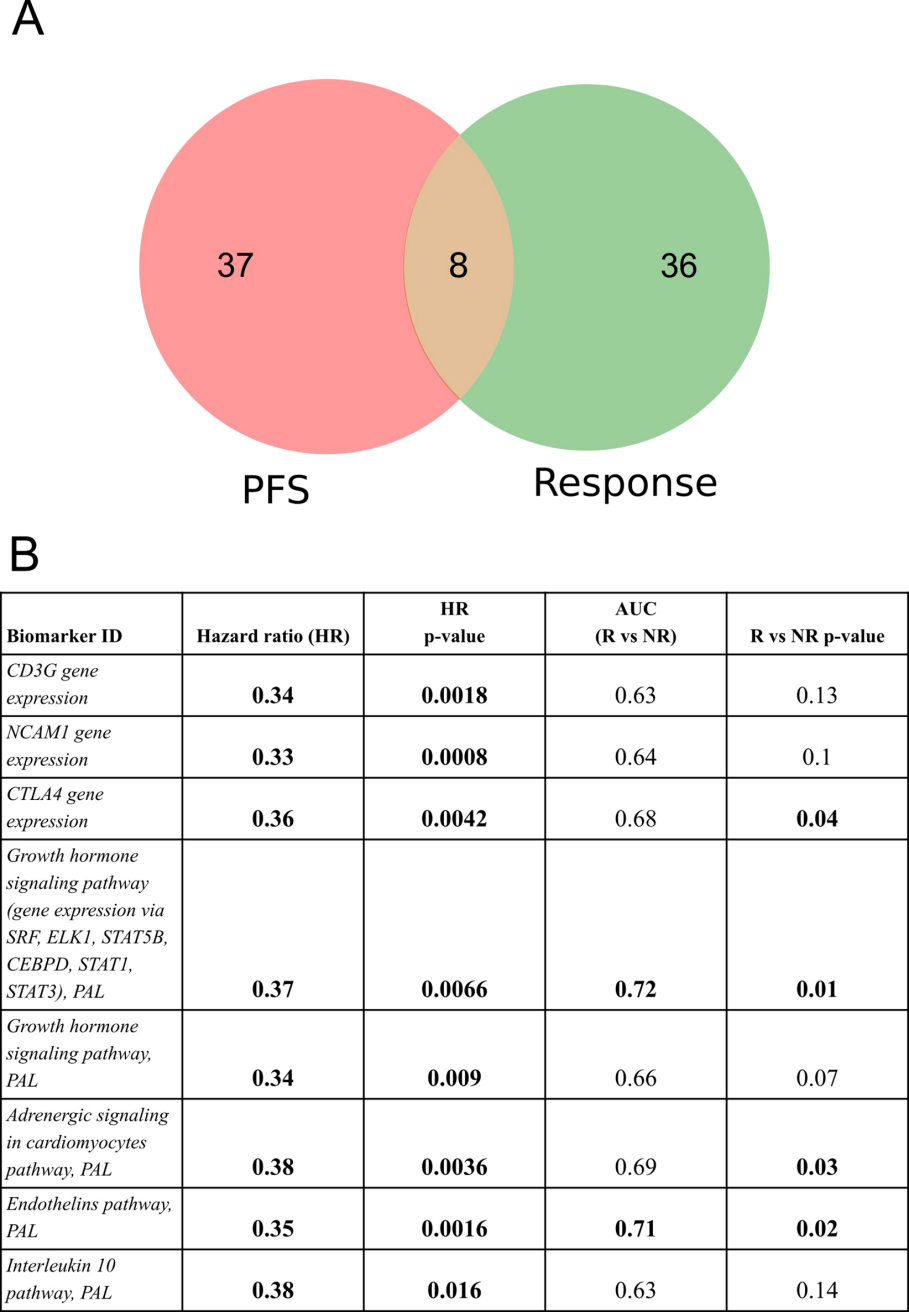
Intersection of PFS biomarkers and RECIST response biomarkers identified in this study for NSCLC patients in relation to responsiveness to PD(L)1 ICI therapeutics. **(A)** Intersection diagram of PFS and RECIST response biomarkers. **(B)** Statistics for the intersected biomarkers identified including PFS-based HR values with *p-values*, AUC values for differentiating treatment responders and non-responders (R vs NR), and Mann-Whitney test *p-values* for differentiating treatment responders and non-responders.

In order to identify a fraction of universal biomarkers that distinguish NSCLC patients by PFS times and RECIST response, we then interested the above 45 PFS biomarkers and 44 RECIST response biomarkers (**Figure 4**).

The following intersected biomarkers were found: (1) PAL of Growth hormone signaling pathway (gene expression via SRF, ELK1, STAT5B, CEBPD, STAT1, STAT3), (2) PAL of Endothelins main pathway, (3) PAL of Adrenergic signaling in cardiomyocytes main pathway, (4) expression level of *CTLA4* gene, (5) PAL of Growth hormone signaling pathway, (6) expression level of *NCAM1* gene, (7) expression level of *CD3G* gene, and (8) PAL of IL10 pathway (**Figure 4**, **Table 2**).

**Table 2:** Major characteristics of intersected PFS and RECIST response biomarkers identified in the experimental NSCLC cohort*.

Biomarker ID	Hazard ratio (HR)	HR *p-value*	AUC (R vs NR)	R vs NR *p-value*
Whole NSCLC cohort				
*CD3G* gene expression	**0.34**	**0.0018**	0.63	0.13
*NCAM1* gene expression	**0.33**	**0.0008**	0.64	0.1
*CTLA4* gene expression	**0.36**	**0.0042**	0.68	**0.04**
*Growth hormone signaling pathway (gene expression via SRF, ELK1, STAT5B, CEBPD, STAT1, STAT3)*, PAL	**0.37**	**0.0066**	**0.72**	**0.01**
*Growth hormone signaling pathway*, PAL	**0.34**	**0.009**	0.66	0.07
*Adrenergic signaling in cardiomyocytes pathway*, PAL	**0.38**	**0.0036**	0.69	**0.03**
*Endothelins pathway*, PAL	**0.35**	**0.0016**	**0.71**	**0.02**
*Interleukin 10 pathway*, PAL	**0.38**	**0.016**	0.63	0.14
Lung adenocarcinoma cohort				
*CD3G* gene expression	**0.21**	**0.022**	**0.82**	0.09
*NCAM1* gene expression	**0.12**	**0.0008**	**0.76**	0.18
*CTLA4* gene expression	**0.17**	**0.001**	**0.9**	**0.03**
*Growth hormone signaling pathway*, PAL	0.44	0.22	0.63	0.55
*Growth hormone signaling pathway (gene expression via SRF, ELK1, STAT5B, CEBPD, STAT1, STAT3)*, PAL	0.41	0.18	0.73	0.26
*Adrenergic signaling in cardiomyocytes pathway*, PAL	0.82	0.72	0.61	0.62
*Endothelins pathway*, PAL	**0.33**	**0.044**	0.53	0.92
*Interleukin 10 pathway*, PAL	0.37	0.21	0.65	0.48
Squamous cell lung carcinoma cohort				
*CD3G* gene expression	**0.33**	**0.026**	0.57	0.58
*NCAM1* gene expression	0.57	0.25	0.64	0.26
*CTLA4* gene expression	0.45	0.091	0.52	0.9
*Growth hormone signaling pathway*, PAL	0.34	0.06	0.72	0.08
*Growth hormone signaling pathway (gene expression via SRF, ELK1, STAT5B, CEBPD, STAT1, STAT3)*, PAL	0.43	0.08	0.7	0.11
*Adrenergic signaling in cardiomyocytes pathway*, PAL	**0.19**	**0.038**	**0.82**	**0.01**
*Endothelins pathway*, PAL	0.48	0.13	0.87	**<0.01**
*Interleukin 10 pathway*, PAL	**0.33**	**0.039**	0.63	0.31

*Significant values are marked with bold font.

**Figure 5:**
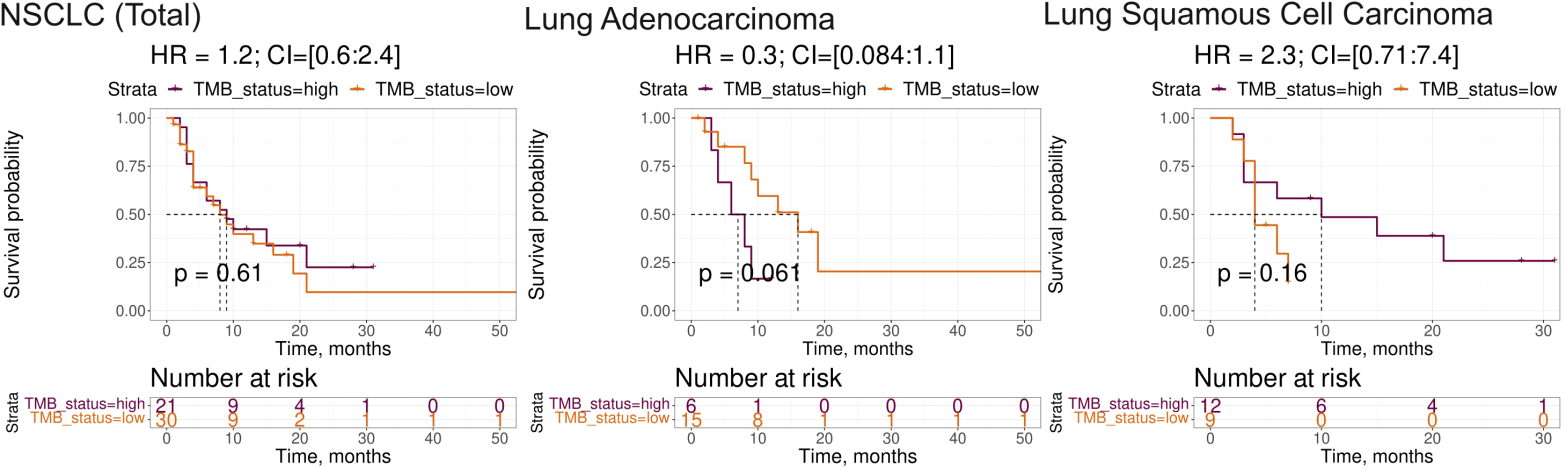
Progression-free survival analysis of tumor mutation burden (TMB) as the biomarker in the experimental NSCLC sampling. The Kaplan-Meier plots are given for the whole NSCLC dataset, and separately for the lung adenocarcinoma and squamous cell lung carcinoma sub-datasets. TMB-high status was defined as TMB greater than 10 per megabase.

Note that among the intersected items there were no WES-derived biomarkers. In our sampling, the well-established NSCLC biomarkers such as TMB and expression level of PD-L1 (*CD274*) gene showed insufficient capacity of distinguishing patients by PFS and/or by RECIST treatment response (**Supplementary Table 3**). In particular, expression level of *CD274* gene showed AUC 0.67 for the whole NSCLC sampling, AUC 0.69 and 0.68 for the lung adenocarcinoma and squamous cell lung carcinoma subsets, respectively. At the same time, in all our cohorts it showed no statistically significant association with the PFS. In turn, TMB showed no significant association with RECIST response except for the squamous cell lung carcinoma subset (AUC 0.72) whereas no statistically significant association with PFS could be detected (**Figure 5**).

The association of TMB and homozygosity of HLA genes was also of an insufficient significance in our samplings (data not shown).

**Figure 6:**
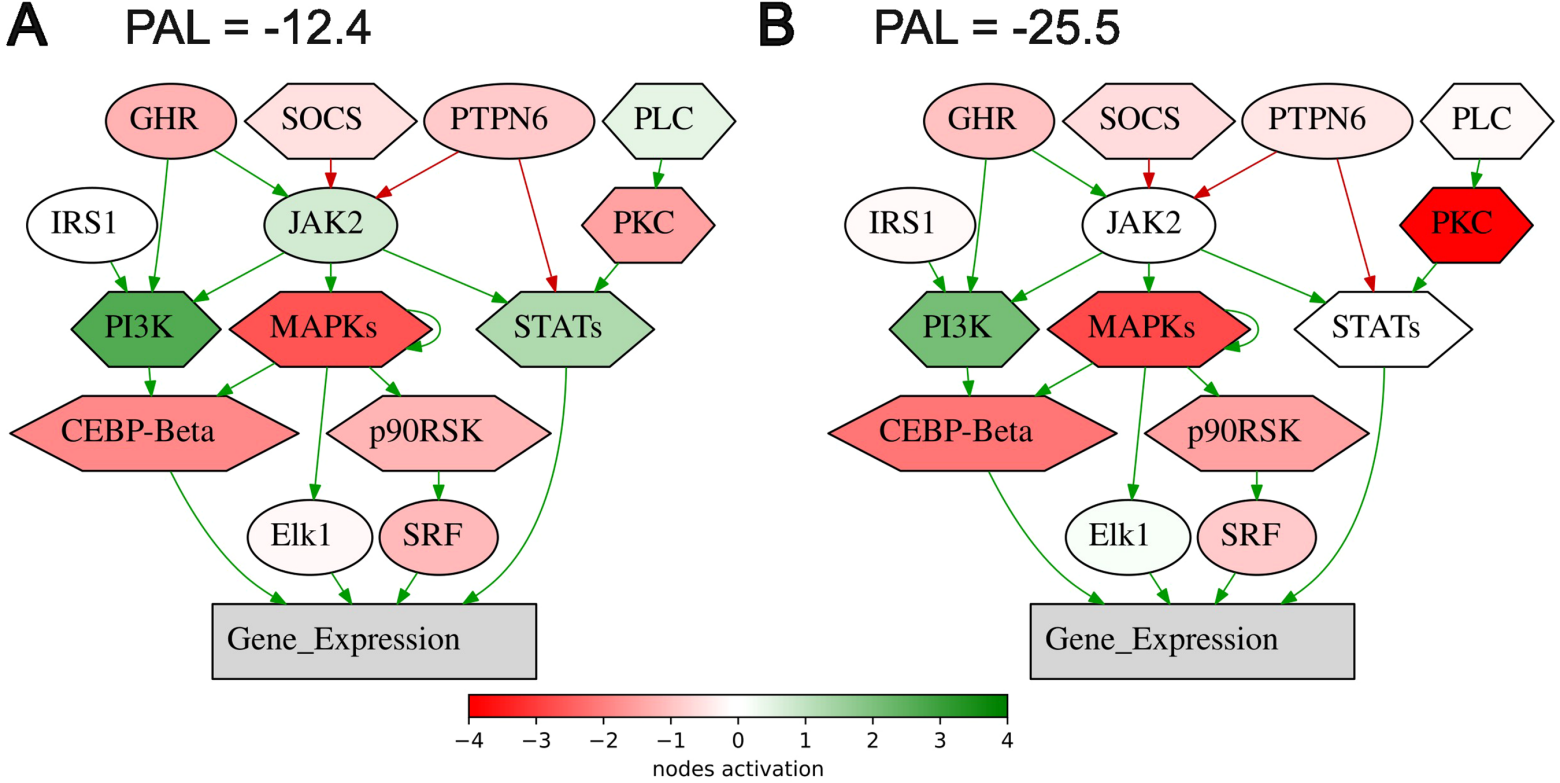
Activation profile of the Growth hormone signaling pathway (gene expression via SRF, ELK1, STAT5B, CEBPD, STAT1, STAT3) in the experimental NSCLC groups of RECIST responders **(A)** and non-responders **(B)** to PD(L)1 ICI immunotherapy. Color reflects the logarithm of the case-to-normal ratio (CNR) of the pathway nodes, color scale is given (green – upregulated, red – downregulated, white – intact). Arrows show molecular interactions within a pathway: green stands for activation, red for inhibition. PAL values were calculated for the averaged biosamples in the treatment responder and non-responder groups.

On the other hand, all of the intersected biomarkers were previously associated with the response to ICI immunotherapies. As to the first biomarker (PAL of *Growth hormone signaling pathway (gene expression via SRF, ELK1, STAT5B, CEBPD, STAT1, STAT3)*, **Figure 6**), growth hormone signaling has been previously associated with the local activity of immune system (52, 53) and with the efficacy of ICI treatment in human cancers (54, 55).

For the second intersected biomarker identified here (PAL of *Endothelins main pathway*), the association of endothelin receptor type B with the response of lung adenocarcinomas to immunotherapy was recently reported (56). In stomach adenocarcinoma, endothelin receptor type A was strongly implicated in building tumor immune microenvironment (57). The third biomarker (PAL of *Adrenergic signaling in cardiomyocytes main pathway*) is also in line with the previous reports of the use of beta-blockers as the supplement to ICI immunotherapy. A trend was observed towards better outcomes in advanced NSCLC patients in the beta-blocker group (median overall survival of 9.93 months in the group not taking beta-blockers versus 14.90 months in the beta-blocker group) (58).

**Figure 7:**
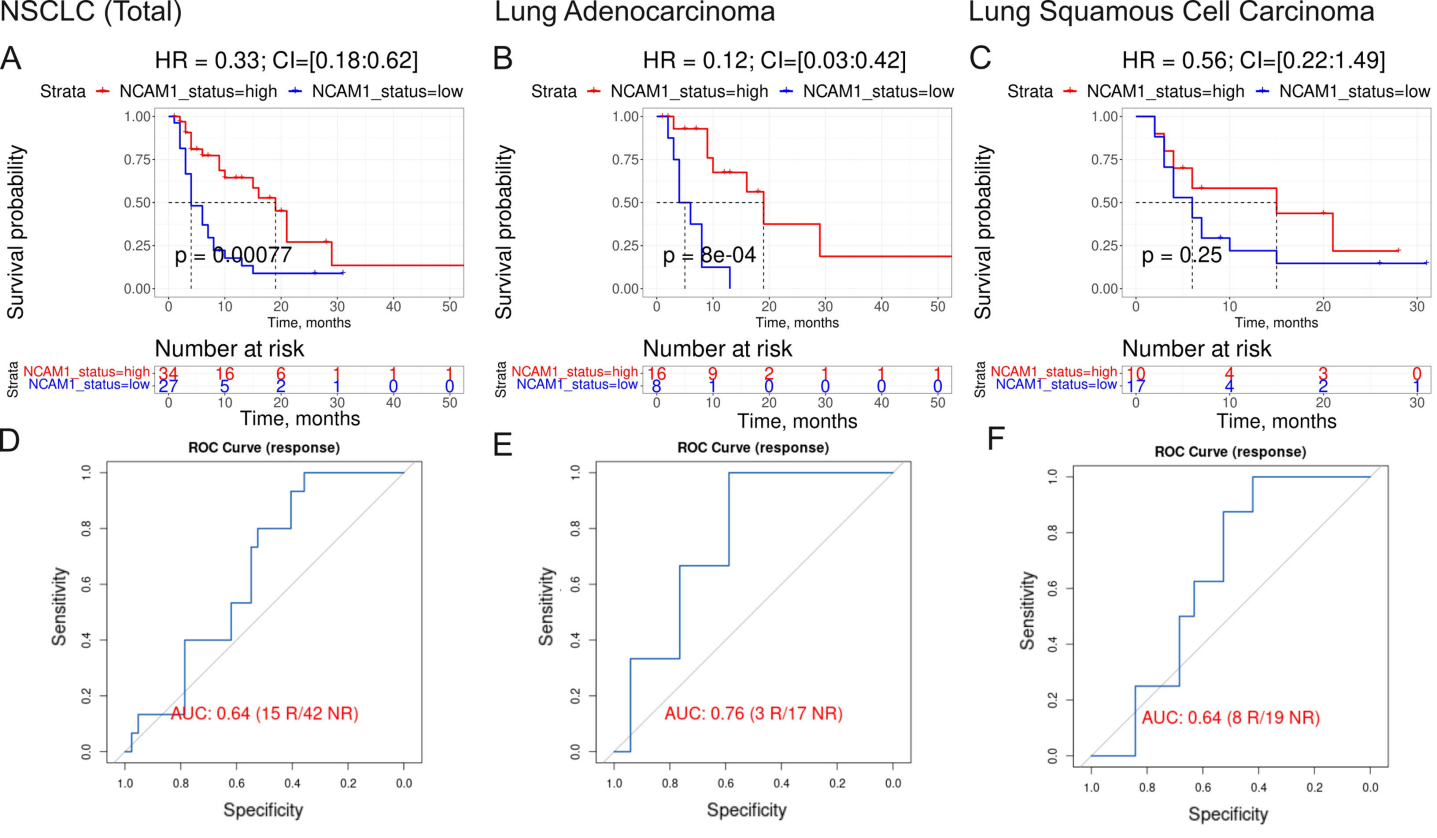
Assessment of biomarker potential of *NCAM1* gene expression level as the PD(L)1 ICI response biomarker in the experimental NSCLC sampling. **(A–C)** Progression-free survival analysis in the experimental NSCLC sampling. The Kaplan-Meier plots are given for the whole NSCLC **(A)** dataset, and separately for the lung adenocarcinoma **(B)** and squamous cell lung carcinoma **(C)** sub-datasets. **(D–F)**, ROC AUC analysis of RECIST response status assessed for the whole NSCLC cohort **(D)** dataset, and separately for the lung adenocarcinoma **(E)** and squamous cell lung carcinoma **(F)** sub-datasets. “R” means treatment responder, “NR” – non-responder.

The fourth biomarker was the expression of *CTLA4* gene which association with ICI immunotherapy is obvious. The fifth biomarker (PAL of *Growth hormone signaling pathway*) was also related to growth hormone pathway which association with ICI immunotherapy is discussed above. The association of the sixth biomarker (expression level of *NCAM1* gene, **Figure 7**) with the response to ICI immunotherapy in lung cancer was also previously reported (59).

**Figure 8:**
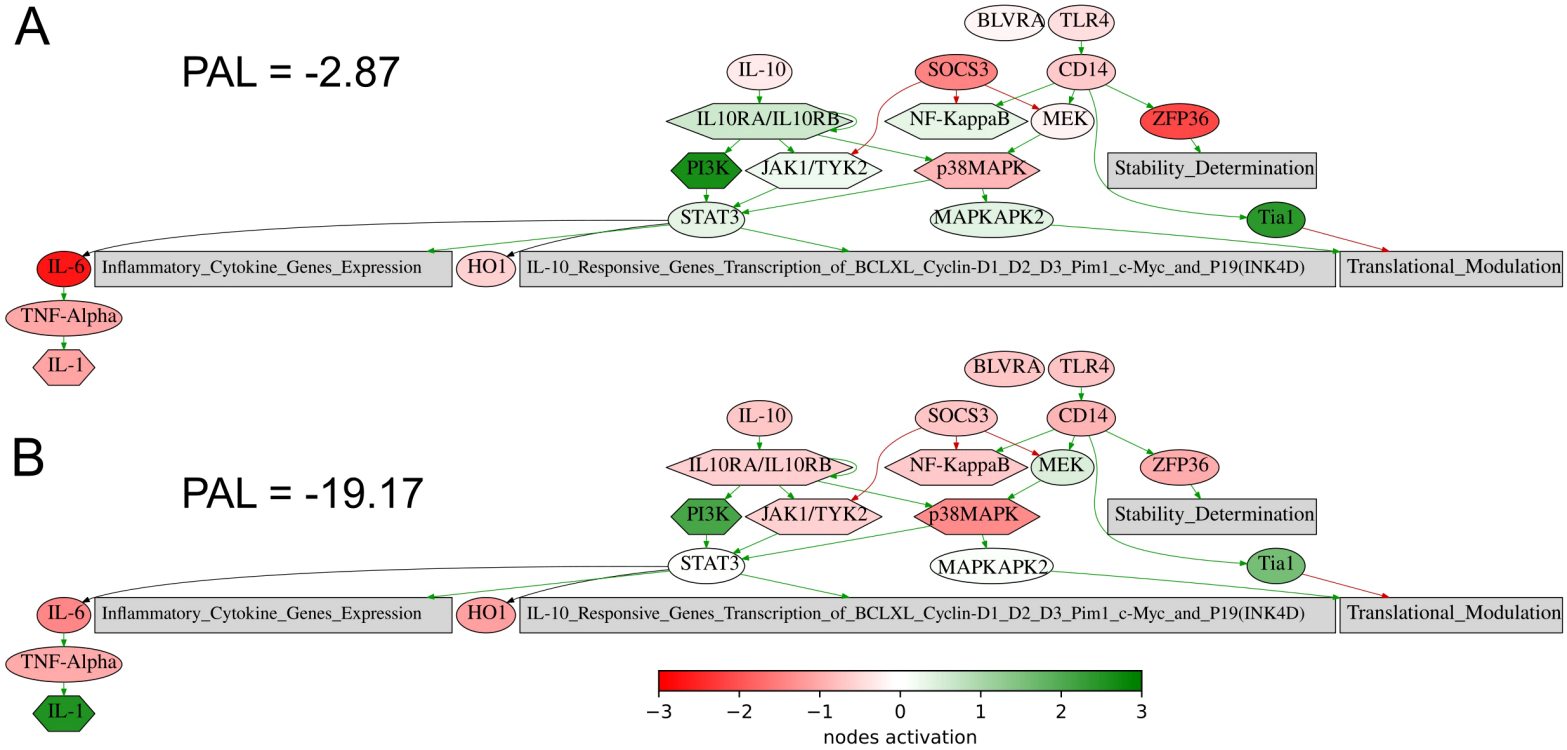
Activation profile of the Interleukin 10 signaling pathway in the experimental NSCLC groups of RECIST responders **(A)** and non-responders **(B)** to PD(L)1 ICI immunotherapy. Color reflects the logarithm of the case-to-normal ratio (CNR) of the pathway nodes, color scale is given (green – upregulated, red – downregulated, white – intact). Arrows show molecular interactions within a pathway: green stands for activation, red for inhibition. PAL values were calculated for the averaged biosamples in the treatment responder and non-responder groups.

The same is true also for the seventh intersected biomarker from our study, expression level of *CD3G* gene, which was found to be associated with better response to lung cancer in a recent report on a lower sampling (60), in line with the current findings. Similarly, for the eighth (last) biomarker, PAL of *IL10 pathway*, **Figure 8**, an association of the level of plasma IL10 with the ICI treatment response in melanoma and NSCLC was recently established (61).

Thus, the intersected biomarkers of NSCLC response on ICI treatment identified here are strongly relevant to immunotherapy efficacy according to the current literature.

### Generation of diagnostic signature of NSCLC response to ICI treatment

Among the intersected PFS- and RECIST-response biomarkers identified on the previous step only two had AUC for discriminating treatment responders and non-responders exceeding 0.7, which is typically considered a threshold for the high-quality biomarkers (**Table 2**). However, none of them could meet the condition of high-quality association with PFS (HR<0.4 or >2.5; *p*<0.05) and AUC exceeding 0.7 for any of the major NSCLC sub-cohorts (adenocarcinoma or squamous cell lung carcinoma, **Table 2**). Thus, we attempted to construct a new ICI response biomarker diagnostic signature that could meet the above quality criteria for at least the whole NSCLC cohort and for one of the above sub-cohorts.

For gene signature generation, we took the above eight intersected biomarkers as the starting components. All biomarker values were normalized using a min-max scaler to ensure equal contribution from each marker in the subsequent analysis. We aimed to design a risk score which is capable of both predicting response and pathological free survival. To this end we combined stepwise HR optimization with multivariate logistic regression model trained to predict patient response.

Our algorithm consisted of following steps:

1. Remove 1 marker from the current set of biomarkers.
2. Predict coefficients for risk score using multivariate logistic regression model.
3. Adjust threshold for the risk score to predict survival as it was done for single markers.
4. Calculate HR for risk score using Cox model.
5. Compare calculated HR with previously acquired HRs.

Marker which removal resulted in best risk score improvement in terms of HR was then removed from the current set of biomarker components. The algorithm was repeated until removal of any component did not result in signature improvement and the ability to distinguish patients with different PFS. Five of the above eight putative biomarkers were incorporated into the final signature (termed Oncobox signature) and were included in the final risk score formula (**Table 3**).

**Table 3:** The components and their multivariate regression logistic coefficients used to calculate the ICI response diagnostic signature-based risk score.

Biomarker component	Regression coefficient
*CD3G* gene expression	-1.79
*NCAM1* gene expression	-2.33
*Adrenergic signaling in cardiomyocytes pathway*, PAL	1.22
*Endothelins main pathway*, PAL	-3.95
*Growth hormone signaling pathway (gene expression via SRF, ELK1, STAT5B, CEBPD, STAT1, STAT3)*, PAL	-7.18

**Figure 9:**
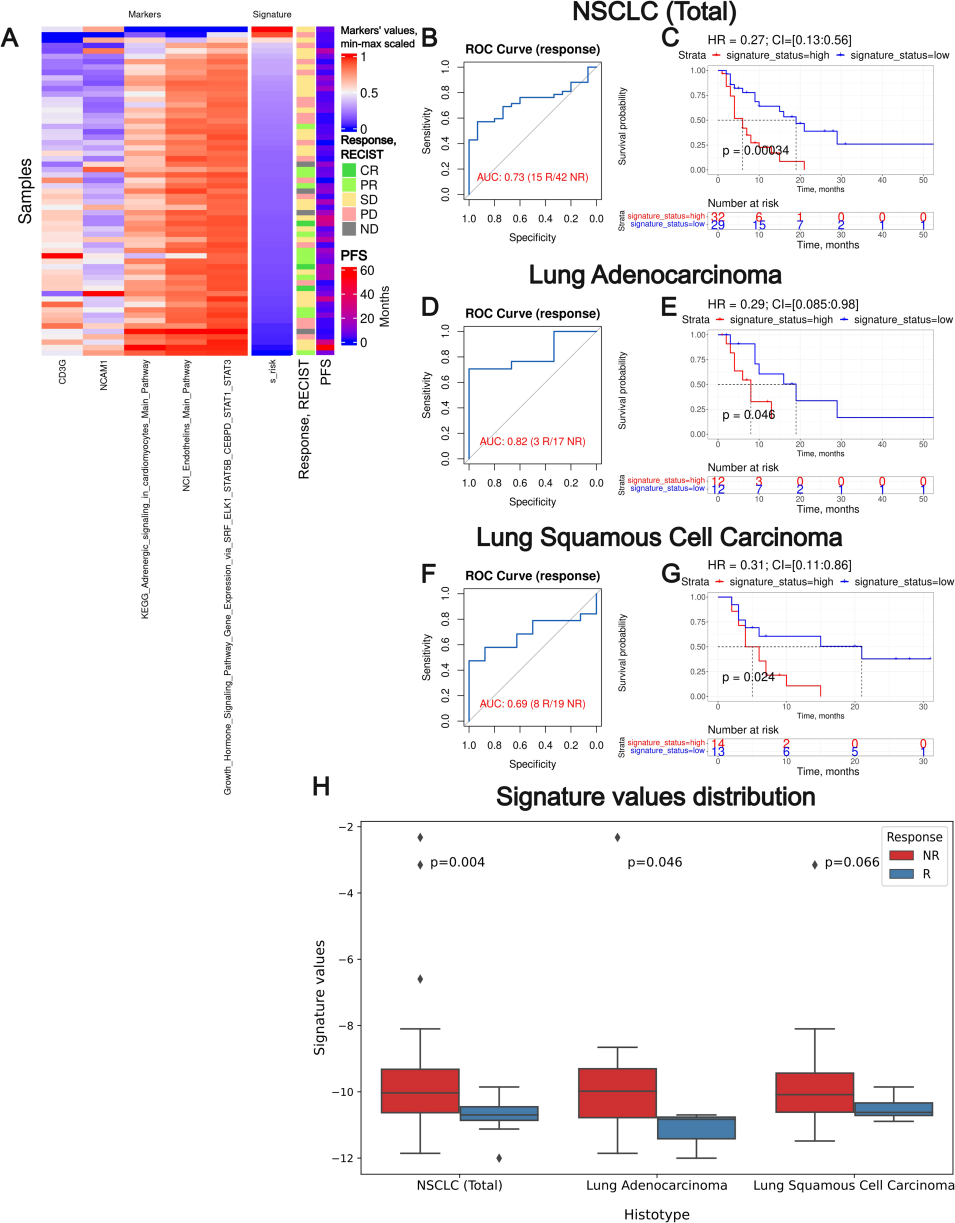
Assessment of biomarker potential of the Oncobox gene signature as the PD(L)1 ICI response biomarker in the experimental NSCLC sampling. **(A)** heatmap outlining normalized values of signature components, signature risk score, response statuses, and PFS times. **(B, D)** *F*, ROC AUC analysis of RECIST response status assessed for the whole NSCLC cohort (*B*) and separately for the lung adenocarcinoma **(D)** and squamous cell lung carcinoma **(F)** sub-cohorts. **(C, E, G)** Progression-free survival analysis in the experimental NSCLC sampling. The Kaplan-Meier plots are given for the whole NSCLC **(C)** dataset, and separately for the lung adenocarcinoma **(E)** and squamous cell lung carcinoma **(G)** sub-datasets. “R” means treatment responder, “NR” – non-responder. **(H)** Box-and-whisker plot for signature values in a whole experimental dataset and in sub-datasets of lung adenocarcinoma and squamous cell carcinoma. p-values are presented for one-sided Mann-Whitney tests between responders (R) and non-responders (NR).

A prognostic risk score of the Oncobox diagnostic signature obtained in this way was calculated using 5 components as a sum of biomarker values multiplied by the coefficients obtained (**Table 3**). We initially assessed the prognostic significance of the Oncobox signature on the experimental NSCLC dataset and two sub-datasets (**Figure 9**). We assessed the Oncobox signature capacity to distinguish patients according to RECIST responder and non-responder outcomes, as well as to group patients by PFS times.

We observed a superior performance for the Oncobox signature compared to any of the individual intersected biomarkers used as the signature components. In all three experimental NSCLC groups, the signature showed strong HR values (0.27-0.31, *p-value* 0.00034-0.046). In two cohorts (whole NSCLC cohort and lung adenocarcinoma cohorts) the signature showed high ROC AUC values (0.73 and 0.82, respectively), whereas in the squamous cell carcinoma cohort the signature showed a borderline value of AUC 0.69 (**Figure 9**).

We also assessed the Oncobox signature performance on a modified experimental cohort with metastatic samples excluded, since their gene expression patterns may be strongly different from the primary tumor sites. The signature preserved its predictive power in terms of response prediction and improved it for prediction of PFS, reaching HR = 0.22 on the whole NSCLC cohort (**Supplementary Figure 1**).

To additionally test the predictive capacity of our signature we applied multivariable survival analysis with variables such as signature status, gender, age and therapy regime. We found that the additional regressors had either non-significant HR values or non-statistically significant p-values, or both, with the exception of a parameter “anti-PD-1 therapy” in lung squamous cell carcinoma (**Supplementary Table 4**), where the smallest HR of 0.06 was assigned to anti PD-1 therapy status. We speculate that this may be due to a strong class imbalance in the above cohort where only one out of 27 patients did not receive anti PD-1 ICI therapy (**Table 1**, **Supplementary Table 1**).

Thus, the Oncobox signature generated here could effectively serve as the biomarker of clinical response to PD(L)1 ICI immunotherapeutics in NSCLC. We then attempted to validate this signature on an independent clinically annotated lung cancer gene expression dataset.

### Validation of Oncobox signature

To validate the diagnostic signature generated, we, first, utilized the publicly available clinically annotated dataset GSE207422 published by Hu and coauthors (62), including RNA sequencing profiles obtained from lung cancer patients who had undergone PD-1 ICI immunotherapy. This independent validation dataset includes 39 samples, 24 of which were collected before the start of ICI immunotherapy and were included in our analysis. We extracted raw sequencing data and the bulk RNAseq FASTQ files were aligned to the hg38 reference genome using the STAR aligner (version 2.7.4a) and gene expression was quantified using Salmon (version 1.3.0) to calculate transcripts per million reads per kilobase (TPM) expression values for each gene.

**Figure 10:**
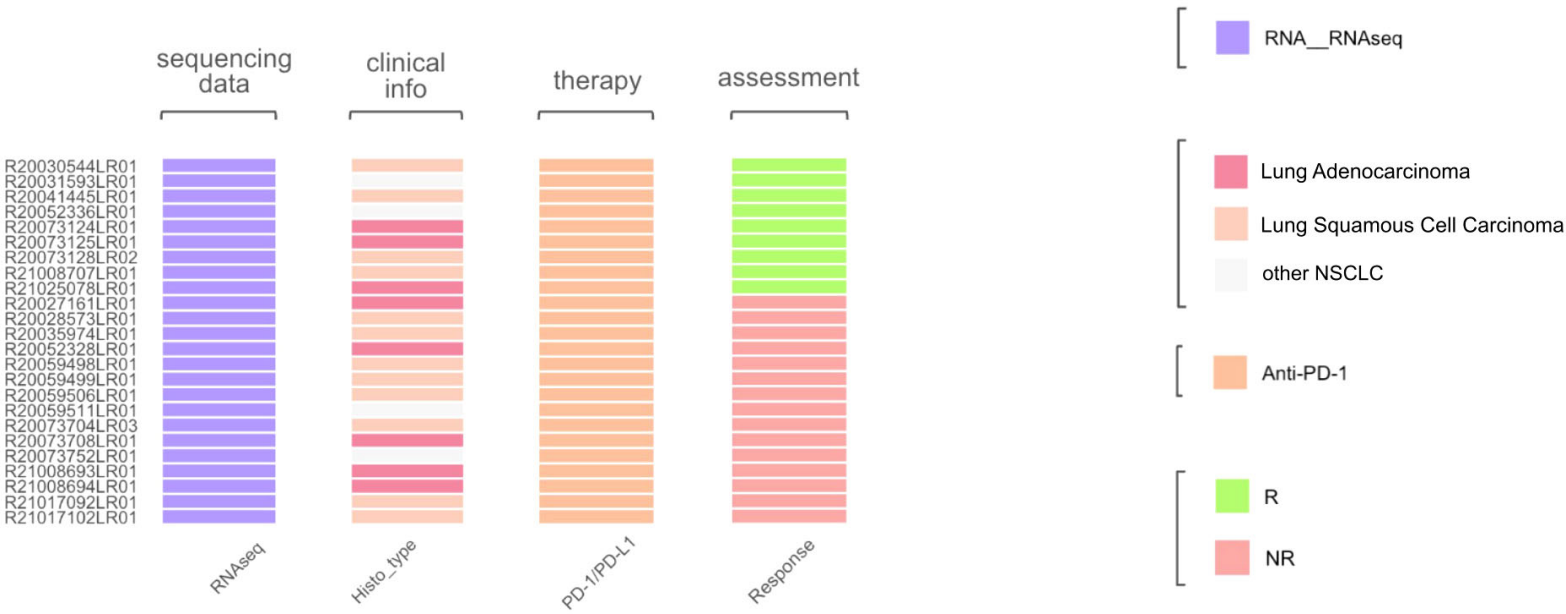
Characteristics of biosamples of the GSE207422 cohort. Sample IDs are given on the left. Color markers indicate availability of the RNAseq profiles; tumor Histotypes established by pathologists; treatment with PD-1 ICI immunotherapeutics; availability of treatment response data (R means treatment responder and NR – non-responder).

Histologically, the selected dataset contained lung adenocarcinoma samples (n = 8) and squamous cell carcinomas (n = 12), along with other NSCLC histotypes. The initial assessment of response to treatment has categorized outcomes as either *major pathological response (MPR*) or *non-MPR*. For consistency, these were reclassified here as the treatment responders and non-responders, respectively (**Figure 10**). No PFS or overall survival data were available.

**Figure 11:**
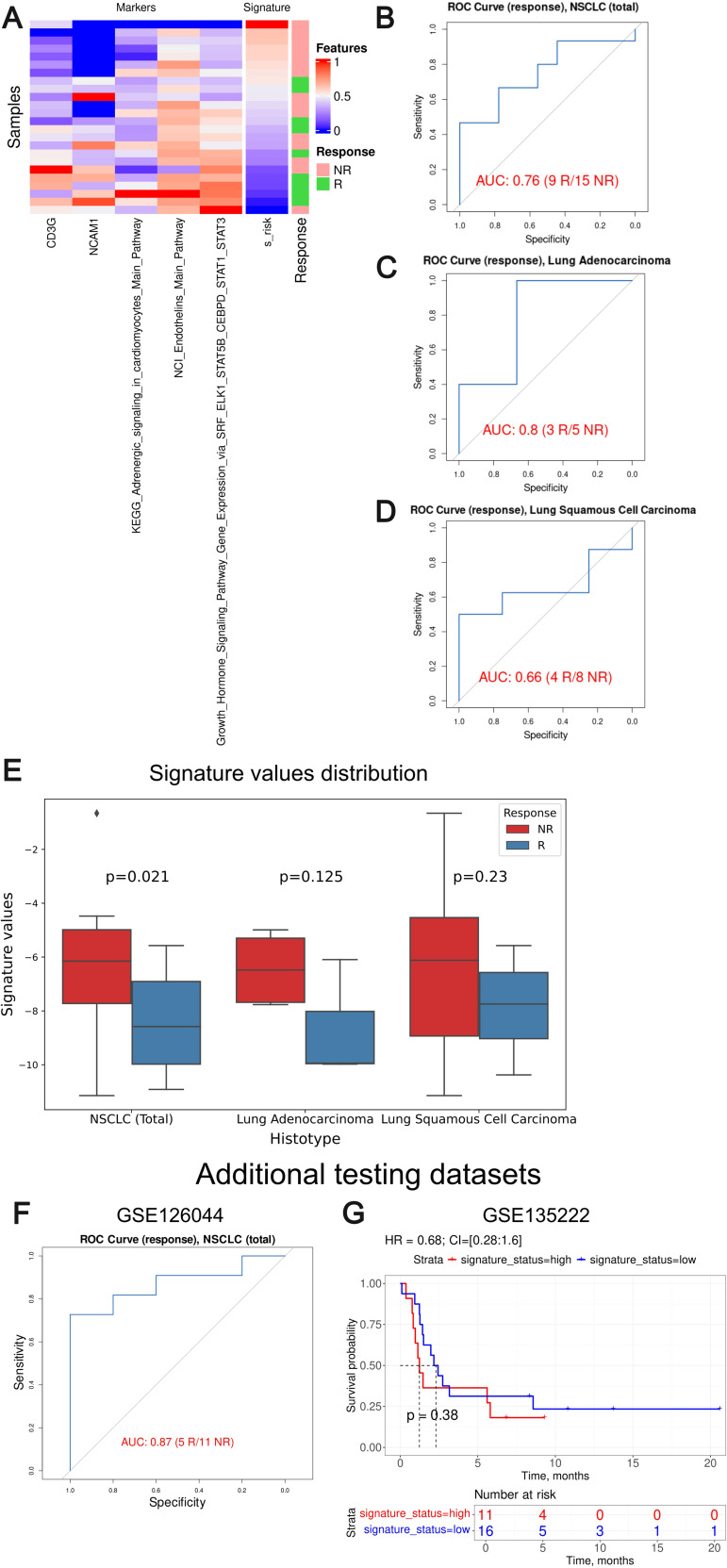
Assessment of biomarker potential of the Oncobox gene signature as the PD-1 ICI response biomarker in the literature GSE207422, GSE126044 and GSE135222 NSCLC cohort. **(A)** Heatmap outlining normalized values of signature components, signature risk score, and response statuses. **(B–D)** ROC AUC analysis of PD-1 immunotherapy response status assessed for the whole GSE207422 NSCLC cohort **(B)**, and separately for the lung adenocarcinoma **(C)** and squamous cell lung carcinoma **(D)** sub-cohorts. “R” means treatment responder, “NR” – non-responder. **(E)** Box-and-whisker plot for signature values in a whole test dataset and in sub-datasets of lung adenocarcinoma and squamous cell carcinoma of GSE207422 dataset. p-values are presented for one-sided Mann-Whitney tests between responders (R) and non-responders (NR) in GSE207422 dataset. **(F)** ROC AUC analysis of PD-(L)1 immunotherapy response status assessed for the GSE126044 NSCLC cohort. **(G)** Progression-free survival analysis in the GSE135222 dataset. The Kaplan-Meier plots are given for the whole NSCLC.

We then tested Oncobox signature risk score on an independent dataset GSE207422. Association with PFS or overall survival was not possible, so we could only assess the response prediction capacity of the signature developed (**Figure 11**). For the whole GSE207422 NSCLC cohort, the Oncobox signature has demonstrated ROC AUC 0.76; for the lung adenocarcinoma cohort, AUC was 0.8. Finally, for the squamous cell carcinoma cohort, AUC 0.66 was registered.

To further validate the performance of our signature we included two additional NSCLC validation datasets: GSE126044 (63) and GSE135222 (64). GSE126044 dataset includes gene expression data for 16 samples obtained before anti-PD-1 therapy (5 treatment responders and 11 non-responders). The clinical data available was the RECIST response status of the patients enrolled. The raw counts were processed in the same way as for the experimental Oncobox dataset. The signature risk values calculated showed AUC of 0.87 for the Oncobox signature, thus confirming its strong predictive capacity (**Figure 11F**).

The dataset GSE135222 included gene expression profiles for samples obtained from 27 NSCLC patients before treatment with anti PD-1/PD-L1 ICI drugs, annotated with PFS times. We calculated signature risk values using transcript-per-million (TPM)-normalized gene expression data available and observed non-significant HR 0.68 with p-value = 0.38, although an overall trend was preserved (**Figure 11G**). We speculate that non-significant results in the latter case may be due to outstandingly small PFS times assigned to the second validation dataset with a median of only 2 months, whereas in the experimental cohort median PFS was three times longer, 6 months.

Thus, we conclude that the Oncobox signature could also distinguish between the PD-1 ICI treatment responders and non-responders in the literature datasets GSE207422 and GSE126044. In two literature and one experimental cohorts, the signature showed high AUC above 0.7 for the whole NSCLC cohort (0.87, 0.76 and 0.73, respectively) and for the adenocarcinoma cohort (0.8 and 0.82). At the same time, borderline values were obtained for the squamous cell carcinoma patients (0.66 and 0.69). However, in the GSE135222 dataset with available PFS records no statistically significant performance was demonstrated which may be connected with the peculiarly short PFS times in the latter cohort.

## Discussion

ICI therapy in lung cancer treatment has gained leading positions along with the approved targeted, chemo-, and radiation therapy methods (65, 66). However, the currently used ICI response biomarkers remain contradictory (4). Thus, more international studies on diverse lung cancer cohorts are needed to identify reliable biomarkers that would be effective in different groups of patients. However, there is a significant gap in the availability of relevant molecular data deposited in the public domain. For example, for this study we were able to find only a modest amount of samples available from 3 datasets (62–64) totaling 67 patients overall, with PFS data available only for GSE126044.

Here we provide a collection of 61 new NSLC molecular profiles clinically annotated by both RECIST response status and by PFS times measured in a longitudinal prospective investigation in relation to a success of treatment by PD-1 and PD-L1 specific ICI therapeutics. The information was deposited to the GEO repository and is fully publicly accessible. All the samples were obtained before the start of PD-(L)1-specific ICI treatment.

Tumor biosamples profiled here were obtained prior to ICI treatment and profiled by whole exome and RNA sequencing. We screened 403 putative biomarkers, including TMB and number of cancer neoantigens, 131 specific HLA alleles, homozygous state of 11 HLA alleles and their superfamilies; four gene mutation biomarkers, expression of 45 immune checkpoint genes and closely related genes and 3 signatures; for the first time, activation levels of 188 molecular pathways containing immune checkpoint genes and activation levels of 19 pathways algorithmically generated using a human interactome model centered around immune checkpoint genes.

We evaluated the potential of biomarkers based on PFS and RECIST treatment response data. In our sample, 45 biomarkers were statistically significantly associated with PFS and 44 with response to treatment, of which eight were shared. Using five of these intersected biomarkers we generated a signature termed Oncobox that showed an AUC of 0.73 and HR of 0.27 (p=0.00034) on the entire experimental NSCLC cohort. This signature was also reliable (AUC 0.76 and 0.87 respectively) for the independent clinically annotated dataset GSE207422 and GSE126044. In both experimental and literature datasets with histotypes markdown available, the Oncobox signature worked better for lung adenocarcinoma than for squamous cell LC. Despite the solid success of response prediction, PFS prediction on the test dataset GSE126044 had reached only modest statistical parameters. However, this might be explained by possible technical differences in PFS reporting as we discussed above.

In our experimental cohorts, 54 patients were treated with PD1-specific and 7 patients with PD-L1-specific ICIs. The PD-1 specific therapeutics were pembrolizumab, nivolumab, and tislelizumab (**Supplementary Table 1**). The PD-L1 immunotherapeutic was avelumab (**Supplementary Table 1**). This set of therapeutics may seem heterogenous in their names, and this is one of the limitations of the current study. However, all of them have the same biological nature (monoclonal humanized therapeutic antibodies), similar molecular mechanism of action and the same FDA-approved molecular biomarkers. Furthermore, this limitation is on the other hand an advantage of the current study as it attempted to uniformly assess molecular biomarkers targeted against the whole PD-1 – PD-L1 signaling axis. Another possible limitation of our study is the inclusion of 12 metastatic samples in the statistical analysis. Theoretically, metastatic samples may preserve genetic features of original tumors; on the other hand, their biology and gene expression patterns can be dramatically different. Nevertheless, we showed here that the exclusion of metastatic samples did not negatively affect the signature performance.

Also, the treatment response status composition of our experimental cohort is unbalanced, especially in the case of Lung Adenocarcinoma subcohort, where among 20 patients only 3 were assigned as the responders, all of these tree samples being of metastatic origins. However, as we demonstrated here, our signature showed good predictive capacity also on the two independent validation datasets. Additional limitation of our study is in the unavailability of protocol data for PD-L1 immunohistochemical status assessment.

More generally, in this study we were limited by the list of pre-selected putative biomarkers and the experimental techniques used. Thus, some putative biomarkers may be missing in our analysis. However, the primary experimental data available here enable to the research community to further explore genetic and transcriptomic biomarkers of tumor ICI responsiveness.

In the future, repertoire of potential biomarkers integrated in one study could be expanded to the simultaneous analysis of tumor proteomic profiles, post-translational modifications, metabolome (67), and the T- and B-cellular composition and receptor repertoire (68).

We hope that the results obtained and the available clinically annotated molecular data will be useful to the research community, especially to those interested in the mechanisms of resistance to ICI therapeutics, and in cancer biomarker research.

## Conclusion

We provide a collection of 61 new NSLC molecular profiles clinically annotated by both RECIST response status and by PFS times measured in a longitudinal prospective investigation in relation to a success of treatment by PD-1 and PD-L1 specific immunotherapeutics. The information is fully publicly accessible. Tumor biosamples were obtained prior to immunotherapy and profiled by whole exome and RNA sequencing. We totally screened 403 putative molecular biomarkers and evaluated their potential based on PFS and RECIST treatment response data. 45 biomarkers were statistically significantly associated with PFS and 44 with response to treatment, of which eight were shared. Using five of these intersected biomarkers we generated a signature termed Oncobox that showed an AUC of 0.73 and HR of 0.27 (p=0.00034) on the entire experimental cohort and AUCs 0.76 and 0.87 on independent literature NSCLC cohorts GSE207422 and GSE126044. For the independent dataset GSE126044 with PFS data available our signature preserved the desirable trend yet demonstrating insignificant predictive power. In both experimental and literature datasets, the Oncobox signature worked better for lung adenocarcinoma than for squamous cell LC.

## Data Availability

The RNA sequencing data are freely accessible through the GEO repository with ID GSE274975. The rest of the relevant data is contained within the article and the supplementary materials.
