# Supplementary material for: Biomarkers of success of anti-PD-(L)1 immunotherapy for non-small cell lung cancer derived from RNA- and whole-exome sequencing: results of a prospective observational study on a cohort of 85 patients
Source: Front Immunol. 2024 Dec 12;15:1493877. doi: 10.3389/fimmu.2024.1493877 (PMC11669362; doi:10.3389/fimmu.2024.1493877)
Supplement: Supplementary file 1 [file Image1.pdf]

NSCLC (Total)

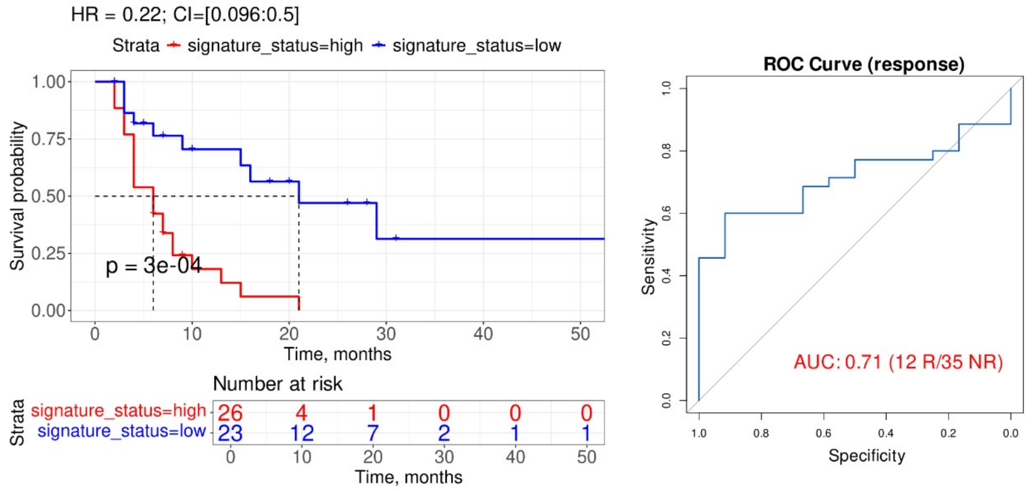

Lung Adenocarcinoma

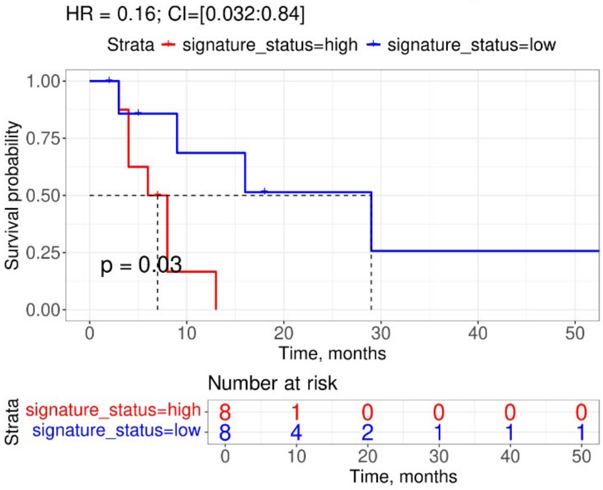

Lung Squamous Cell Carcinoma

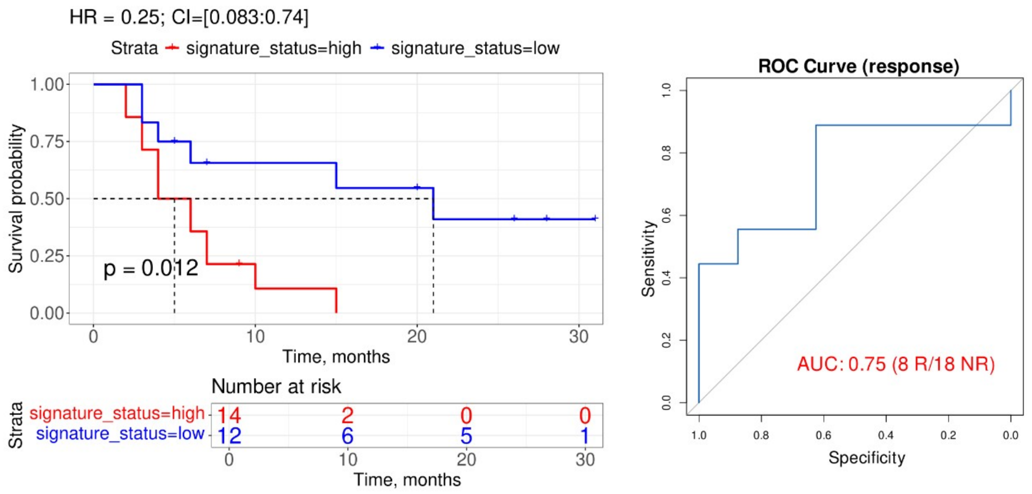

Supplementary figure 1. Signature parameters on Oncobox datasets with excluded metastatic samples
